# SeizFt: Interpretable Machine Learning for Seizure Detection Using Wearables

**DOI:** 10.3390/bioengineering10080918

**Published:** 2023-08-02

**Authors:** Irfan Al-Hussaini, Cassie S. Mitchell

**Affiliations:** 1 School of Electrical and Computer Engineering, Georgia Institute of Technology, Atlanta, GA 30332, USA; 2 Department of Biomedical Engineering, Georgia Institute of Technology and Emory University, Atlanta, GA 30332, USA; 3 Machine Learning Center at Georgia Tech, Georgia Institute of Technology, Atlanta, GA 30332, USA

**Keywords:** seizure, EEG, augmentation, xai, interpretability, imbalanced classes, electroencephalogram, artificial intelligence, machine learning

## Abstract

This work presents SeizFt—a novel seizure detection framework that utilizes machine learning to automatically detect seizures using wearable SensorDot EEG data. Inspired by interpretable sleep staging, our novel approach employs a unique combination of data augmentation, meaningful feature extraction, and an ensemble of decision trees to improve resilience to variations in EEG and to increase the capacity to generalize to unseen data. Fourier Transform (FT) Surrogates were utilized to increase sample size and improve the class balance between labeled non-seizure and seizure epochs. To enhance model stability and accuracy, SeizFt utilizes an ensemble of decision trees through the CatBoost classifier to classify each second of EEG recording as seizure or non-seizure. The SeizIt1 dataset was used for training, and the SeizIt2 dataset for validation and testing. Model performance for seizure detection was evaluated using two primary metrics: sensitivity using the any-overlap method (OVLP) and False Alarm (FA) rate using epoch-based scoring (EPOCH). Notably, SeizFt placed first among an array of state-of-the-art seizure detection algorithms as part of the Seizure Detection Grand Challenge at the 2023 International Conference on Acoustics, Speech, and Signal Processing (ICASSP). SeizFt outperformed state-of-the-art black-box models in accurate seizure detection and minimized false alarms, obtaining a total score of 40.15, combining OVLP and EPOCH across two tasks and representing an improvement of ~30% from the next best approach. The interpretability of SeizFt is a key advantage, as it fosters trust and accountability among healthcare professionals. The most predictive seizure detection features extracted from SeizFt were: delta wave, interquartile range, standard deviation, total absolute power, theta wave, the ratio of delta to theta, binned entropy, Hjorth complexity, delta + theta, and Higuchi fractal dimension. In conclusion, the successful application of SeizFt to wearable SensorDot data suggests its potential for real-time, continuous monitoring to improve personalized medicine for epilepsy.

## 1. Introduction

Seizures are abnormal, uncontrolled electrical discharges in the brain that can manifest as various symptoms ranging from mild to severe [1,2]. Common seizure symptoms include involuntary muscle movements, partial to full loss of consciousness, and cognitive disturbances, especially in the ictal and post-ictal phases [3,4]. Seizure events are particularly relevant for individuals suffering from epilepsy, a chronic neurological disorder characterized by recurrent seizures. The timely and accurate detection of seizures is critical for the appropriate diagnosis and management of epilepsy. Seizure detection using wearables enables healthcare professionals to devise tailored treatment plans and monitor the efficacy of therapeutic interventions [5,6,7,8]. Furthermore, the identification of seizure patterns can help prevent potential injury and improve the overall quality of life of epilepsy patients [9,10,11].

In clinical practice, the gold standard for seizure detection is the Electroencephalogram (EEG). The EEG is a non-invasive technique that records electrical activity in the brain via electrodes placed on the scalp in a specific pattern that correlates with the lobes of the brain. Trained clinical neurophysiologists analyze the EEG recordings to identify specific patterns indicative of seizures and to attempt to localize the originating location of the seizure activity [12,13,14]. However, this manual approach is time-consuming, requires extensive expertise, and can be prone to human error, particularly when analyzing very large volumes of data. Moreover, the financial burden associated with conducting and interpreting EEGs further highlights the need for more efficient and cost-effective methods.

Machine learning (ML) and artificial intelligence (AI) have the potential to revolutionize the field of seizure detection by automating the analysis of EEG recordings, thereby drastically reducing the associated time and cost [15,16]. By leveraging advanced algorithms capable of identifying complex patterns in large datasets [17,18,19,20,21], ML-based systems can achieve high levels of accuracy. In fact, in some instances, ML surpasses human performance. Recently developed wearable EEG devices are more portable and patient-friendly. For example, behind-the-ear electrodes can enable continuous, real-time monitoring of brain activity for prompt intervention and optimal epilepsy management [22,23,24,25].

In recent years, deep learning methodologies have demonstrated significant potential for effective seizure detection [26,27,28,29]. Deep learning is a subset of machine learning that employs artificial neural networks with many hidden layers. Such artificial neural networks learn hierarchical representations of input data, which makes them particularly suitable for analyzing complex, high-dimensional EEG signals [30,31,32,33,34]. Some deep learning models have obtained remarkable accuracy for detecting seizures, such as Convolutional Neural Network (CNN) [28], Recurrent-CNN (RCNN) [26], and auto-encoders [27]. However, deep learning models often lack interpretability, which is crucial for fostering trust and accountability in clinical settings [35,36,37,38,39].

The presented SeizFt work expands upon research conducted as part of the Seizure Detection Challenge 2023 (https://signalprocessingsociety.org/publications-resources/data-challenges/seizure-detection-challenge-icassp-2023 (accessed on 30 July 2023)), a Grand Challenge at the International Conference on Acoustics, Speech, and Signal Processing (ICASSP). The Grand Challenge, which is detailed in Section 2, aimed to develop advanced machine learning frameworks capable of accurately detecting seizures in patients with epilepsy using EEG data collected from a discreet wearable device equipped with behind-the-ear electrodes. The Grand Challenge was divided into two distinct tasks, each targeting a different aspect of seizure detection: *Task 1* focused on the development of a machine learning model for detecting seizures in wearable SensorDot (SD) data, while *Task 2* centered around the optimization of a given Deep Learning model for wearable seizure detection.

The SeizFt framework aims to revolutionize epilepsy management by offering a more efficient, accurate, and interpretable approach to seizure detection. By combining robust features, data augmentation, class balancing, and an interpretable machine learning algorithm, we demonstrate the feasibility of developing advanced seizure detection systems that can significantly improve the quality of life for individuals living with epilepsy. The SeizFt approach addresses the need for transparency and understandability while maintaining high levels of accuracy.

The SeizFt framework leverages a variety of robust features extracted from the EEG data that were inspired by recent work in interpretable sleep staging [39,40]. These features include Standard Deviation (STD), Inter-Quartile Range (IQR), Skewness, Kurtosis, Number of Zero Crossings, Hjorth mobility and complexity, Fractal dimensions, Entropies, and the Power in Different Energy Bands, such as Delta. Such features provide comprehensive information about the underlying characteristics of the EEG signals that enable the model to make more accurate and interpretable predictions.

Data augmentation and class balancing play a crucial role in improving the performance of our SeizFt framework. In this study, we employ Fourier Transform (FT) Surrogates [41,42] to augment the EEG signals during training and balance the number of seizure and non-seizure epochs. This technique significantly helps in addressing the challenges associated with imbalanced datasets, which are commonly found in medical applications. For example, in continuous epilepsy monitoring, seizure events are very rare compared to non-seizure epochs. Moreover, the augmentation strategy enhances the generalization capabilities of the model. As such, the model still performs well on unseen data.

The SeizFt framework is built upon an ensemble of trees using CatBoost [43,44]. The ensemble approach combines the predictions of multiple trees, leading to a more accurate and robust model. Additionally, CatBoost effectively handles class imbalance by assigning weights to the classes during training, which further improves the model’s performance in detecting seizures.

In our experiments, we demonstrate the effectiveness of the SeizFt framework. SeizFt is compared with other state-of-the-art approaches, such as ChronoNet [45] and a proposed deep neural network with multi-headed attention, referred to as AttentionNet [46]. Results illustrate that SeizFt achieves superior sensitivity and lower false alarm rates on the SeizeIT2 dataset [47], resulting in an overall improvement of 30% from state-of-the-art benchmarks. Utilizing our proposed SeizFt framework for Task 1 and our presented augmentation and class balancing strategy for Task 2, we obtained scores of 47.57 and 29.01, respectively, as evaluated on the holdout test, SeizeIT2 [47] provided by the challenge administrators. These combined efforts resulted in a total score of 40.15, marking our submission as the only submitted method that exceeded the established benchmark of 31.03, which was set by the Grand Challenge organizers. Finally, we provide an interpretation of the most important features used by the SeizFt model, which offers valuable insights into the characteristics of seizures as captured by EEG signals.

In summary, this paper makes several key contributions to the field, as outlined below:

- First, we propose a deep learning model that combines CNN, Long Short-Term Memory (LSTM), and multi-headed attention elements outperforming traditional deep learning methods.
- We propose SeizFt, a robust and explainable seizure detection method for wearable EEG devices. This framework merges several computational strategies, including feature extraction, data augmentation via Fourier Transform (FT) Surrogates, class balancing, and a CatBoost-driven ensemble of decision trees.
- By means of comprehensive experimental analysis, we validate the superior performance of SeizFt. We show that our model excels in terms of sensitivity and false alarm rates, consistently outperforming other established state-of-the-art methods.
- Importantly, we highlight the vital, clinically interpretable features that SeizFt employs to characterize seizures as captured by EEG. These critical features underline the interpretability of our model, enhancing trust in its predictive ability and marking a significant advance in the integration of machine learning within a clinical context.
- Finally, we consider the practical implications and future potential of SeizFt, asserting that it establishes a new benchmark for seizure detection using wearable EEG and suggest possible directions for future research and application. This, we believe, will inspire advancements that could profoundly impact patient care.

## 2. The 2023 ICASSP Seizure Detection Grand Challenge

This presented work for SeizFt represents the winning submission to the Seizure Detection Challenge 2023, a Grand Challenge hosted by the International Conference on Acoustics, Speech and Signal Processing (ICASSP). This section details the Grand Challenge tasks and datasets, which inspired the development of SeizFt.

### 2.1. Tasks

There were two core tasks: Seizure Detection and Data-Centric Seizure Detection. Additionally, two specific datasets were provided, as described below.

The *Seizure Detection* task (Task 1) required the creation of a machine learning (ML) model to identify seizures using wearable SD data. For training this model, we used the SeizeIT1 dataset [48]. However, the test set comprised data gathered through the wearable SD device. We had the option, but not the obligation, to leverage the full scalp EEG (vEEG) data available in the training set. The pre-trained model was built to encompass routines employed for pre and post-processing. We fed the model with wearable EEG and/or single-channel ECG data from the SD device. The final aim was to assign a seizure or non-seizure label for each second in the recording.

The second task, *Data-Centric Seizure Detection* (Task 2), concentrated on the ’data-centric AI’ approach. This task emphasized the significance of data quality and the representation of a variety of cases within the training set for optimizing seizure detection performance. Unlike Task 1, the goal here was to apply data manipulation techniques to enhance the performance of the model provided for wearable seizure detection. For this task, we utilized the same training set as in Task 1 and were given a Deep Learning model—an adapted version of the ChronoNet [45]. This model was designed to accept a two-second, two-channel EEG window resampled at a sampling frequency of 200 Hz. The model was expected to output a binary vector where each consecutive two-second EEG segment was marked as a seizure or non-seizure period.

### 2.2. Data Sources

The data used in this study is sourced from two EEG datasets—*SeizeIt1* [48] and *SeizeIT2* [47]. Summaries of the data utilized in this study are provided below. For additional details, please refer to the datasets’ originally published works [47,48].

#### 2.2.1. SeizeIt1 Dataset—Training Set

The SeizeIt1 [48] dataset was collected during an ICON project (2017–2018) through collaboration between KU Leuven (ESAT-STADIUS), UZ Leuven, UCB, Byteflies, and Pilipili. The goal of the project was to design a home environment patient monitoring system using behind-the-ear (bhe) EEG electrodes. The data were collected in the hospital during presurgical evaluation. The dataset includes a full 10–20 scalp EEG data, behind-the-ear data, and single-lead ECG data. Seizure annotations were performed by clinicians based on the gold standard vEEG system.

The data of 82 patients were recorded between 23 January 2017 and 26 October 2018. Of these patients, 54 were recorded with the behind-the-ear channels. In total, 42 of those patients experienced seizures during their presurgical evaluation. The number of seizures per patient ranged from 1 to 22, with a median of 3 seizures per patient. The duration of the seizures, the time difference of seizure EEG onset and end, varied between 11 and 695 s with a median of 50 s. In all, 89% of the seizures were Focal Impaired Awareness seizures, and 91% of the seizures originated from the (fronto-) temporal lobe.

The dataset made available to 2023 ICASSP Seizure Detection Grand Challenge participants contained data from the 42 patients who experienced epileptic events during the recording period.

#### 2.2.2. SeizeIT2 Dataset—Validation and Test Set

The SeizeIT2 dataset, an extension of the SeizeIT1 project, contains EEG and ECG recordings of more than 350 patients with epilepsy, making use of a wearable Seizure Detection (SD) device for in-home and hospital seizure monitoring. It represents the first phase-4 clinical trial of wearable in-home monitoring for seizure detection. It brings together public and private stakeholders, including academic institutions such as Université de Navarra, Karolinska Institutet, KU Leuven, RWTH Aachen, Universitäts klinikum Aachen, Centro Hospitalar e Universitário de Coimbra, Oxford University Hospitals NHS Foundation Trust, and Stockholms Läns Landsting, led by UCB. The focus of this dataset is the clinical validation of the SD in patients experiencing typical absence, focal impaired awareness, and generalized tonic-clonic seizures. Patients underwent video-EEG monitoring with additional electrodes attached behind each ear for concomitant SD recording. The SD device was secured on the upper back using a patch, and the impedance was maintained at ≤5 kΩ.

In the context of the 2023 ICASSP Seizure Detection Grand Challenge, the SensorDot data of the patients in UZ Leuven was used for the test set, as well as for the validation set. The SensorDot recordings from 2 patients were provided to the participants to validate the performance of their models (Table 1). All the models were tested by the Grand Challenge organizers in the remaining 31 adult patients from UZ Leuven who had focal seizures (Table 2).

### 2.3. Performance Metrics

In seizure detection, sensitivity and false alarm (FA) rates are typically prioritized as performance metrics due to the seizure event’s rarity. These metrics offer a more precise measure of the model’s ability to correctly identify true seizure events, which is crucial. For SeizFt, model performance is evaluated using two primary metrics: Sensitivity using the any-overlap method (OVLP) [49] and False Alarm (FA) rate using epoch-based scoring (EPOCH) [49]. Sensitivity measures the proportion of correctly identified seizure epochs, while the FA rate quantifies the number of false alarms per hour. To balance sensitivity and FA rate, a weighting factor of −0.4 is applied. The overall performance score is then calculated as the weighted average of Task 1 and Task 2 scores, with weights of 0.6 for Task 1 and 0.4 for Task 2. The scoring formula is as follows:

(1) $$\begin{array}{cc} {\mathrm{Score}}_{\mathrm{Task}\;x} & ={\mathrm{Sensitivity}}_{\mathrm{Task}\;x}-0.4{\mathrm{FA}}_{\mathrm{Task}\;x}/\mathrm{hour} \end{array}$$

(2) $$\begin{array}{cc} \mathrm{Total}\_\mathrm{Score} & =0.6{\mathrm{Score}}_{\mathrm{Task}\;1}+0.4{\mathrm{Score}}_{\mathrm{Task}\;2} \end{array}$$

Equation (1) serves to compute the separate scores for both Task 1 and Task 2. These independent results are subsequently merged using Equation (2) to deduce the comprehensive total score. As such, it is worth noting that Task 1’s score holds a heavier weight in the determination of the overall score.

## 3. Methods

Here we present the framework, construction, and other methods utilized to develop SeizFt. The specific objective of SeizFt was to surpass deep learning model accuracy with an algorithm that enables transparent analysis of clinically interpretable features. The work was split into two tasks that aligned with the previously stated 2023 ICASSP Seizure Detection Grand Challenge tasks: seizure detection and data-centric seizure detection.

### 3.1. SeizFt Model Framework

**Figure 1:**
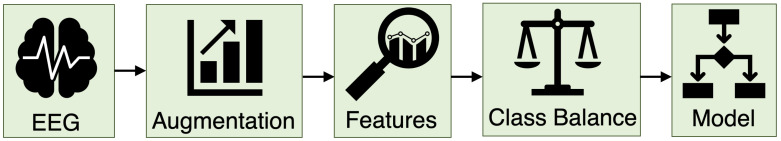
SeizFt Framework.

We propose SeizFt, a framework for interpreting features in seizure detection as part of Task 1. This model’s success hinges on a systematic sequence of steps used during the training process to bolster both the model’s accuracy and its interpretability. The framework is illustrated in Figure 1. Our approach is outlined in several stages:

#### 3.1.1. Data Augmentation and Class Balancing

A common issue in biomedical signal analysis, including EEG signals used in seizure detection, is the quality and quantity of available data. Often, the amount of data is insufficient for training robust machine learning models. Moreover, the data may also be unbalanced. Unbalanced data has far more observations for one label (or class) type compared to another. For example, in epileptic EEG monitoring, there is typically much more data collected during non-seizure periods than during periods of seizure. To mitigate issues with sample size and class imbalance, we employ data augmentation techniques. Data augmentation is a relatively common machine learning technique used to improve model performance and, in particular, to improve the model’s ability to generalize to new, unseen data [50,51,52].

In the context of our work, Fourier Transform (FT) Surrogates are used for data augmentation [41,42]. FT Surrogates is a mathematical technique that transforms a function of time, such as an EEG signal, into a function of frequency. This transformed function, or spectrum, provides a different perspective on the data and highlights different aspects of the underlying signal.

FT Surrogates are a specific type of data augmentation where surrogate data are generated by randomizing the phases of the original EEG signal’s Fourier Transform. Crucially, this is done while preserving the power spectrum, which is a measure of the signal’s power intensity in the frequency domain. This ensures that the overall structure of the original signal is maintained, even as the temporal organization of the signal (e.g., the specific order and timing of events) is altered.

The use of FT Surrogates serves two primary purposes. Firstly, it increases the size of the training dataset. By creating additional, varied examples from the existing data, the model has more material from which to learn, which improves its performance. Secondly, this method adds diversity to the training dataset. The surrogate data are similar to but distinct from, the original data. As such, they allow the model to learn to recognize seizures in a broader range of circumstances.

Moreover, FT Surrogates help to address class imbalance, a common issue in biomedical machine learning. In many datasets, there are more examples of non-seizure periods than seizure periods, which can skew the model’s learning. By generating additional seizure and non-seizure epochs using FT Surrogates, we can balance the distribution of classes in the training data. As a result, the model learns to recognize both seizure and non-seizure periods equally well.

#### 3.1.2. Feature Extraction

This study’s approach to EEG feature extraction is inspired by recent advancements in interpretable sleep staging [39,40]. These advancements have yielded methods for effectively and robustly extracting features from EEG signals that capture the underlying characteristics of the signals. The specific features extracted in the present study are categorized into four main types: statistical measures, temporal features, complexity measures, and spectral features.

Statistical measures used were Standard Deviation (STD), Interquartile Range (IQR), Skewness, and Kurtosis. STD and IQR provide a measure of variability in the EEG signals. STD quantifies the average distance of the data, and IQR provides a measure of statistical dispersion. Skewness and kurtosis describe the shape of the signal’s probability distribution. Skewness measures the asymmetry, and kurtosis assesses the heaviness of the signal’s tails relative to a normal distribution.

Temporal features considered include the Number of Zero Crossings, Hjorth mobility, and complexity. The number of zero crossings provides an indication of the signal’s frequency, while Hjorth parameters (mobility and complexity) are descriptive statistics used to characterize the signal. Specifically, mobility measures the mean frequency or the signal’s rate of change. Complexity compares the signal’s similarity to a pure sine wave.

The complexity measures utilized were Fractal dimensions and Entropies. Fractal dimensions measure how the detail in the data changes with the scale. On the other hand, entropy provides a quantitative measure of the signal’s randomness or unpredictability.

Spectral features, such as power in different energy bands, such as Delta, Theta, Alpha, and Beta, are also extracted. These spectral features divide the total EEG signal into different frequency bands and measure the relative power within each band. These energy bands represent different brain states and activities. For instance, Delta waves are associated with deep, dreamless sleep and regeneration; Theta with creativity and insight; Alpha with relaxed awareness; and Beta with active thinking and focus [53].

The combination of these features presents a comprehensive representation of the EEG signals that enables the model to effectively discern between seizure and non-seizure epochs. We maximize the potential to detect and classify seizure activity within the data by capturing the nuances of the signals through this diverse set of features. The comprehensive and robust feature extraction method forms a strong foundation for the development of an effective seizure detection model.

#### 3.1.3. Model Training

For the *Seizure Detection Task*, or *Task 1*, we used CatBoost [43,44]—a machine learning algorithm based on gradient boosting over decision trees—to classify seizures and non-seizures in each epoch. It is an ensemble learning method that performs exceptionally well across a broad spectrum of tasks by iteratively combining hundreds or thousands of weak learners to generate a powerful single predictive model.

One of the crucial challenges in seizure detection using machine learning is the severe class imbalance typically present in the data. Seizures are rare events and hence, are vastly outnumbered by non-seizure epochs. To address this issue, we integrated weights into the CatBoost model. These weights were derived from the distribution of seizure and non-seizure epochs after the application of the augmentation strategy, FT Surrogate [41].

The *Data-Centric Seizure Detection Task*, or *Task 2*, also faced the problem of class imbalance between seizure and non-seizure epochs. Again, the FT Surrogate methodology was utilized for EEG data augmentation. Moreover, the relative class weights were dynamically updated prior to the model’s training phase. This approach was based on the recognition that the quality of the training data and their ability to represent diverse cases is crucial for the success of machine learning applications. By augmenting the data and recalibrating the class weights, SeizFt created a training set that robustly represents both seizure and non-seizure data.

In essence, this two-pronged approach ensured that the deep learning model was effectively optimized for wearable seizure detection. Simultaneously, it maintained a balance between seizure and non-seizure data representation, allowing for a more reliable and accurate model for seizure detection.

### 3.2. Experimental Setup

Our study employed the SeizeIT1 dataset [48], which comprised EEG data from epilepsy patients that was clearly annotated to indicate seizure and non-seizure epochs. This dataset was subjected to a random split, with an 8:2 ratio, which formed the training and validation sets, respectively.

Our model’s performance was further tested on the SeizeIT2 dataset [47], which acted as an independent test set. This allowed us to assess the model’s generalizability and performance on unseen data. It is important to note that some of the evaluation results detailed in subsequent sections were performed by the challenge organizers, given that we did not have access to a specific held-out test set. This arrangement ensured an unbiased evaluation of our model’s performance.

Several software tools and libraries were employed throughout this study to facilitate various stages of model development. We utilized Braindecode and MNE [42,54] for FT Surrogate augmentation, which is a vital step in augmenting our dataset. Feature extraction, a critical pre-processing step in machine learning, was performed using tsflex [55], scipy [56], and scikit-learn [57]. These libraries provide a comprehensive set of methods for extracting meaningful features from our time-series data. For the training phase, different libraries were used depending on the model. CatBoost [43,44] was chosen to train SeizFt. For training *AttentionNet*, we opted for PyTorch 2.0 [58]. Lastly, TensorFlow [59] was the library of choice for training ChronoNet [45].

### 3.3. Baselines

**Figure 2:**
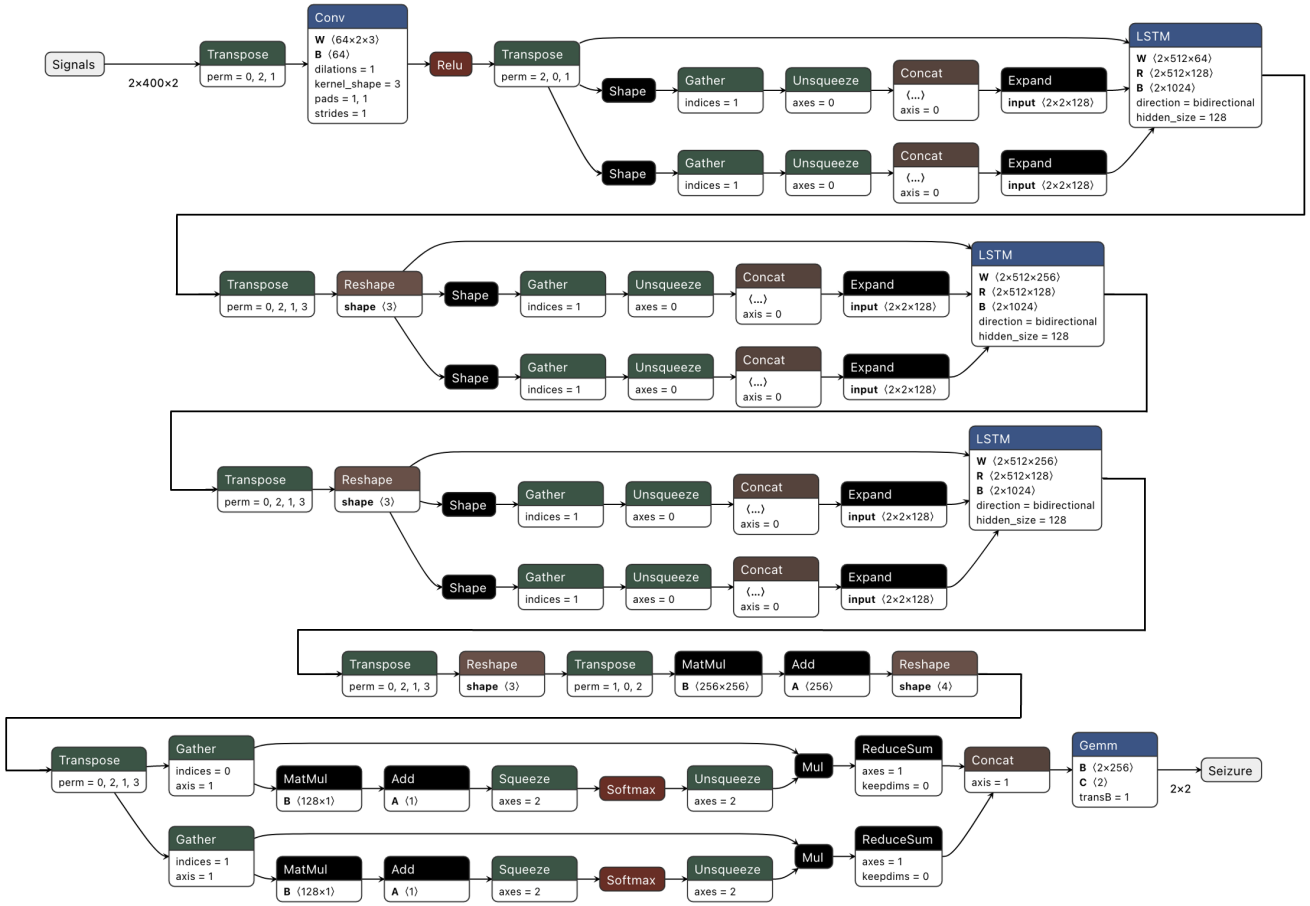
AttentionNet architecture.

The following were used as baselines to compare the performance of SeizFt to other high-performing or state-of-the-art seizure detection models:

- *AttentionNet*: The architecture, as depicted in Figure 2, integrates a neural network comprised of CNN layers followed by LSTM layers. It is augmented with a multi-headed attention mechanism.
- ChronoNet [45]: This approach utilized a novel Recurrent Neural Network (RNN) architecture, dubbed ChronoNet, which was inspired by recent advancements in image classification. The authors adapted the methodology to EEG data interpretation to improve the efficiency and accuracy in distinguishing between normal and abnormal brain activity. ChronoNet incorporates multiple 1D convolution layers followed by deep Gated Recurrent Unit (GRU) layers, taking raw time-series EEG data and learning to identify patterns in brain activity.
- Transformer and CNN [60]: Recognizing the limitation of conventional EEG systems and the need for timely and accurate diagnosis, the authors adopted a transformer-based deep neural network approach. This model incorporated a mixed Transformer and CNN architecture, which was trained and pre-trained on several datasets, including the Temple University Hospital Seizure Corpus [61]. To improve the model’s robustness and mitigate overfitting, dropout, and early stopping techniques were employed. For the second task, the authors enhanced the ChronoNet architecture for abnormal EEG detection by boosting the signal-to-noise ratio in EEG data and optimizing various training parameters.
- Spectral Power and Random Forest [62]: This approach to epilepsy monitoring used lightweight machine-learning models specifically tailored for resource-constrained wearable devices. The framework uses the Random Forest (RF) algorithm in conjunction with power features extracted from specific EEG frequency bands for seizure detection. The power features were derived from different frequency ranges and are calculated through a combination of time-domain band-pass filtering and the application of Parseval’s theorem to the filtered signal. For the RF algorithm, an ensemble of relatively shallow decision trees is employed. The final prediction was obtained by voting on the outcomes of all the trees, which reduces computational complexity for real-time inference on wearable devices. In the second task, the data-centric ChronoNet architecture was utilized to optimize certain hyperparameters to balance seizure detection sensitivity and false alarm rates.
- Deep Convolutional Neural Network [63]: EEG data were preprocessed, standardized, and segmented into 2-second intervals before model training. Experiments were performed with several deep CNN architectures and a range of input types, such as raw EEG signals, Short-time Fourier Transform (STFT) transformed signals, wavelets, and mel scale spectral transformations. Over 100 different DCNN models were trained. The optimal models were chosen based on the mean of lower boundaries of 95% confidence intervals for cross-validation and hold-out scores. Additionally, data augmentation techniques were implemented in the second task to improve the performance of the ChronoNet architecture.

## 4. Results

The performance and interpretability of SeizFt was evaluated and compared to other state-of-the-art automated seizure detection methods.

### 4.1. Comparative Analysis

Table 1 and Table 2 demonstrate the efficacy of the SeizFt approach compared to other high-performing, state-of-the-art methods on the same dataset. A thorough comparative analysis is presented in Table 1, which benchmarks the performance of our proposed SeizFt model against various state-of-the-art models. Notably, these include ChronoNet [45] and the multi-headed attention deep neural network, AttentionNet (refer to Figure 2). The table also features an optimized version of the ChronoNet model specifically designed for Task 2 to showcase the effectiveness of data-driven methodologies in seizure detection tasks.

The SeizFt model, constructed for Task 1, outperforms all other tested models in sensitivity, false alarm rate, and total score. As such, SeizFt sets a new performance benchmark. The commendable performance boost of SeizFt is primarily due to the efficient data augmentation and class balancing techniques adopted. Our proposed approach, which employs the same ChronoNet model as the organizer benchmark (shown in the first row) but adds class re-balancing and FT Surrogates, achieves an approximately threefold improvement over their baseline model, as can be seen in the second row of the table. An optimized ChronoNet model, specifically designed for Task 2 and using a similar methodology, also depicts a substantial reduction in false alarm rate and an increase in total score compared to the baseline ChronoNet [45]. Collectively, these results reaffirm the efficacy of the presented SeizFt methodology.

**Table 1:** Model Evaluation on SeizeIT2 Dataset [47] ${}^{a}$.

	Sensitivity (OVLP [49])	False Alarms per Hour (EPOCH [49])	Total Score
ChronoNet [45]	58.22	117.12	11.37
AttentionNet (Figure 2)	53.57	30.85	41.23
SeizFt (Task 1)	**62.86**	14.93	**56.88**
ChronoNet (Task 2)	22.22	**9.82**	18.30

${}^{a}$ Bold signifies the best score for the corresponding metric.

**Table 2:** Official Results of the ICASSP Seizure Detection Grand Challenge ${}^{a}$.

	Score Task 1	Score Task 2	Total Score
Benchmark	45.10	10.42	31.03
Pathology Dynamics (SeizFt)	**47.57**	29.01	**40.15**
UCLA CDx [60]	26.92	**29.32**	27.88
Neural Engineering Lab [62]	36.98	4.06	23.81
Brainify.ai [63]	6.54	25.21	14.00

${}^{a}$ Bold signifies the best score for the corresponding metric.

The performance of the models was independently verified by the 2023 ICASSP Seizure Detection Grand Challenge’s competition organizers using a concealed test set. This process ensured a rigorous and unbiased evaluation of all models’ generalization capabilities to unseen data. The final confusion matrix output for SeizFt and other model entries using the independent concealed test set was not made available. Instead, the scoring Equations (1) and (2) shown in Section 2 were used to compare model performance and generalizability to the unseen concealed test set. Despite the limited access to the evaluation dataset, the provided comparative analysis illustrates the superior performance of SeizFt over other state-of-the-art methods, including the organizer Benchmark model.

The final evaluation results for the 2023 ICASSP Seizure Detection Grand Challenge are presented in Table 2. Note that our entry in the 2023 ICASSP Seizure Detection Grand Challenge official results was listed under our research laboratory name, “Pathology Dynamics”. To be clear, the listed Pathology Dynamics entry is SeizFt. The SeizFt approach for Task 1 and Task 2 achieves the highest [or best] total score of 40.15. Thus, SeizFt was the only method to surpass the 2023 ICASSP Seizure Detection Grand Challenge organizers’ benchmark, which scored 31.03. This achievement underscores the effectiveness of the SeizFt methodology and its promising potential for practical application in seizure detection.

Notably, the above-described comparative results illustrate the importance of the SeizFt employed Fourier-transform surrogates to address prior limitations of deep learning. Through the randomization of Fourier-transform phases of temporal-spatial data, FT Surrogates are generated that provide a means to balance the dataset for EEG-based seizure detection. The technique utilizes these surrogates to augment underrepresented classes, thereby achieving a more balanced dataset for training subsequent models. Evidence of the impact of the FT Surrogate method can be seen in the improvement of Task 2, as outlined in Table 2. Our proposed approach employs the same ChronoNet model as the organizer Benchmark. However, our approach adds class re-balancing and FT Surrogates. Due to the addition of these surrogates, SeizFt achieved an approximately threefold improvement (shown in line 2 of Table 2) over the organizer Benchmark model (shown in line 1 of Table 2).

### 4.2. Model Interpretation

**Figure 3:**
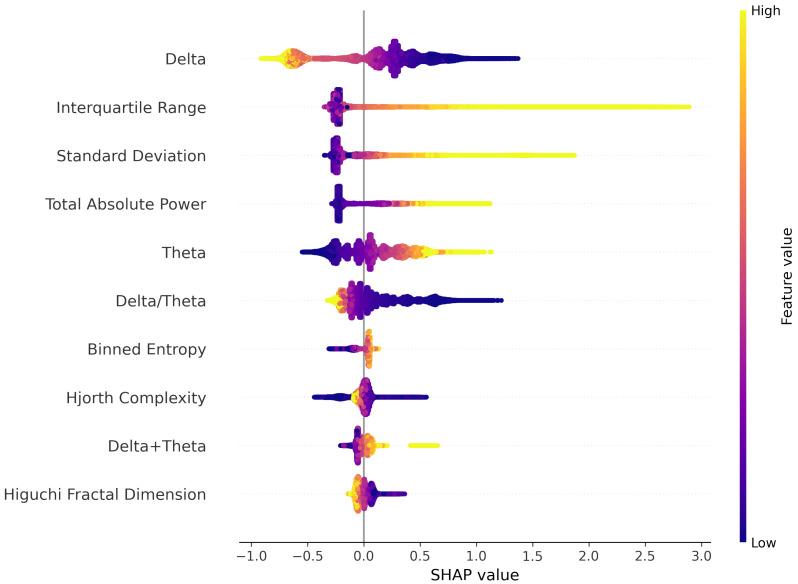
The ten most impactful features according to SHAP for SeizFt [64].

The SeizFt model’s decision-making process relies on several key features. The top ten features are visually represented in Figure 3, as identified by the SHAP method [64]. These results demonstrate the model’s ability to discern the influence of various frequency bands in an EEG signal on seizure detection. Notably, the examination of features from the frequency bands in an EEG signal is a common method for identifying correlations indicative of seizures [65].

The foremost influential feature, representative of the lower delta band within a frequency range of 0.4 to 1 Hz, reveals an intriguing correlation: decreased values within this band could enhance the detection of a seizure. Conversely, the feature associated with the theta band, which spans 4–8 Hz, reveals an inverse relationship. Higher values within this band seem to decrease the likelihood of a seizure prediction by our model. The sixth feature, which highlights the power ratio of the delta band to the theta band, offers a compelling insight: a reduction in this ratio seems to boost the effectiveness of seizure detection. These observations concur with the findings by Shoeb et al. [15], who established a correlation between higher frequencies and seizure epochs.

In accordance with previous research [66], seizure epochs display higher values of both the Interquartile Range and Standard Deviation as compared to non-seizure epochs. Entropy, however, shows similar values across seizure and non-seizure epochs. These observations are confirmed by the 2nd, 3rd, and 7th most impactful features depicted in Figure 3. Adding to these insights, Greene et al. [67] suggest that the Total Absolute Power exhibits an increased value during seizure epochs compared to non-seizure epochs. This observation is supported by the 4th most significant feature and further corroborated by the 9th feature that amalgamates the power in both Delta and Theta bands.

These findings reinforce the ability of the SeizFt model to distinguish accurately between seizure and non-seizure epochs not only in the frequency domain of EEG signals but also in the time domain. This distinction further confirms the potential utility of SeizFt in real-world seizure detection applications.

### 4.3. Model Robustness and Generalizability

The performance of SeizFt on the SeizeIT2 dataset [47] underscores the robust nature of the model in recognizing seizures from wearable SensorDot data. The model’s design incorporates data augmentation and class balancing strategies, which improve its resilience to variations and anomalies in EEG signals and bolsters its capacity to generalize to unseen data. Moreover, SeizFt utilizes an ensemble of decision trees through the CatBoost classifier [43,44], enhancing the model’s stability and accuracy. This strategic choice enables the model to provide reliable and accurate predictions for deployment in clinical scenarios. As such, the development of real-time, on-device seizure prediction algorithms using the SeizFt framework could enable proactive epilepsy management and early intervention strategies.

## 5. Discussion

In this study, we have presented SeizFt, an interpretable seizure detection framework that combines robust feature extraction with an effective augmentation strategy. The main contributions of this study are as follows:

- We propose a deep neural network that incorporates CNN, LSTM, and multi-headed attention elements, which outperforms other deep learning techniques.
- We introduce SeizFt, a robust and interpretable framework for seizure detection in wearable EEGs, which amalgamates feature extraction, data augmentation via Fourier Transform (FT) Surrogates, class balancing, and a CatBoost-driven ensemble of decision trees.
- Through experimental evaluation, we illustrate the superior performance of our SeizFt framework in terms of sensitivity and false alarm rates relative to existing state-of-the-art methods.
- We elucidate the crucial, clinically interpretable features employed by the SeizFt framework that characterize seizures as measured via EEG. The SeizFt features enhance trust in the model’s predictive capability.

### 5.1. Interpretable Models Can Compete with Black-Box Models

Traditionally, deep learning “black-box” models have outperformed their “glass-box” or interpretable model counterparts when it comes to predictive accuracy. However, the lack of interpretability of black-box models to explain their predictions lessens trust in their use for high-stakes healthcare decision support. Ideally, healthcare applications need black-box model accuracy with glass-box model interpretability. Our approach with SeizFt demonstrates that interpretable models can, in fact, outperform black-box deep learning methods for detecting rare events in physiological monitoring. SeizFt was able to eclipse state-of-the-art black-box model accuracy for seizure detection and minimize false alarms. Moreover, SeizFt maintains transparent model interpretability using well-established features trusted by practicing clinicians.

### 5.2. Evaluation of Design Choices That Made SeizFt a Success

With its accuracy besting state-of-the-art deep learning models and its feature-based interpretability, SeizFt becomes a new benchmark for automated seizure detection using wearable EEG devices. The success of SeizFt can be attributed to several key factors.

Firstly, the use of Fourier Transform (FT) Surrogates for data augmentation addresses the challenge of class imbalance, which is commonly encountered in seizure detection tasks. By generating synthetic seizure and non-seizure epochs, our approach ensures a balanced representation of both classes during the training process. The achieved class balance results in a more accurate and generalizable model.

Secondly, SeizFt employs a diverse set of robust features inspired by recent work in interpretable sleep staging [39]. These features, such as standard deviation, interquartile range, skewness, kurtosis, and power in different energy bands, enable the model to capture the complex characteristics of EEG signals associated with seizure events. This, in turn, allows for more accurate classification of seizure and non-seizure epochs.

Finally, the model design choice of employing an ensemble of trees using CatBoost as the classifier plays a crucial role in the effectiveness of SeizFt. The use of an ensemble approach provides increased stability and accuracy in the model, which is essential for its adoption in clinical settings where reliability is of utmost importance.

### 5.3. Potential for Clinical Impact

The proposed SeizFt framework could markedly revolutionize the clinical management of epilepsy, offering the capability for precise, real-time seizure detection through wearable devices. Unlike many black-box machine learning models, SeizFt is an interpretable model. As such, SeizFt fosters trust and transparency in its use in clinical settings [35,37,38]. More specifically, the model bases its predictions on concrete and tangible features. As such, the likelihood of false positives and negatives is reduced, which increases overall reliability. With this framework, healthcare professionals can promptly intervene during an epileptic seizure and adjust treatment plans in a personalized manner. Thus, SeizFt has the potential to significantly improve patient outcomes and enhance the quality of life for individuals living with epilepsy.

### 5.4. Limitations and Future Directions

SeizFt has shown impressive performance in seizure detection through wearable devices, outperforming state-of-the-art benchmarks by ~30%. Although the method demonstrates adaptability to various datasets and a broad range of applications, further trials on more diverse epilepsy datasets and with different wearable EEG sensors would provide a more comprehensive assessment of the method’s efficiency. There is potential for future research to incorporate additional physiological indicators known to fluctuate before, during, and after a seizure. Features such as heart rate variability, motion data, and perspiration [65] could potentially augment the model’s performance in real time. As the technologies in integrated sensors advance, additional features such as changes in environmental light and sound could be considered, given their known potential to precipitate seizures in some individuals.

## 6. Conclusions

In conclusion, our proposed SeizFt framework represents a significant advancement in the field of seizure detection. By leveraging a combination of robust feature extraction, data augmentation, and class balancing strategies, SeizFt demonstrates superior performance compared to other state-of-the-art approaches, including black-box deep learning methods. The interpretability of SeizFt is a key advantage, as it fosters trust and accountability among healthcare professionals who rely on the model to make critical decisions regarding the diagnosis and management of epilepsy. Moreover, the successful application of SeizFt to wearable SensorDot data suggests its potential for real-time, continuous monitoring of brain activity to improve patient diagnostics, management, intervention, and quality of life via personalized medicine.

## Data Availability

The code for SeizFt is freely available on GitHub at: https://github.com/iah3/interpretable-seizure (accessed on 30 July 2023). Data were made available to participants of the 2023 ICASSP Seizure Detection Grand Challenge. Requests for data should be made to the Grand Challenge organizers: https://biomedepi.github.io/seizure_detection_challenge/team/ (accessed on 30 July 2023).
